# Human Body 3D Posture Estimation Using Significant Points and Two Cameras

**DOI:** 10.1155/2014/670953

**Published:** 2014-04-30

**Authors:** Chia-Feng Juang, Teng-Chang Chen, Wei-Chin Du

**Affiliations:** Department of Electrical Engineering, National Chung Hsing University, Taichung 402, Taiwan

## Abstract

This paper proposes a three-dimensional (3D) human posture estimation system that locates 3D significant body points based on 2D body contours extracted from two cameras without using any depth sensors. The 3D significant body points that are located by this system include the head, the center of the body, the tips of the feet, the tips of the hands, the elbows, and the knees. First, a linear support vector machine- (SVM-) based segmentation method is proposed to distinguish the human body from the background in red, green, and blue (RGB) color space. The SVM-based segmentation method uses not only normalized color differences but also included angle between pixels in the current frame and the background in order to reduce shadow influence. After segmentation, 2D significant points in each of the two extracted images are located. A significant point volume matching (SPVM) method is then proposed to reconstruct the 3D significant body point locations by using 2D posture estimation results. Experimental results show that the proposed SVM-based segmentation method shows better performance than other gray level- and RGB-based segmentation approaches. This paper also shows the effectiveness of the 3D posture estimation results in different postures.

## 1. Introduction

The development of a computer vision-based intelligent system for object detection [1–4], understanding human behavior [5–12], or estimating posture [13–22] has attracted research attention in recent years. Research topics on understanding human behavior include human activities monitoring [5–8] and posture classification [9–12], to name just a few. Human body posture estimation is helpful for analyzing, interpreting, and animating human actions. Examples include the animation of realistic human characters with computer graphics in animated cartoons [13], virtual space teleconferencing [14], and analysis of the actions of sports players [15]. Vision-based 3D posture estimation approaches can be divided into two categories, marker-based [16, 17] and marker-free [18–22]. For marker-based approaches, a set of distinguishable color markers are attached to the significant body points, like head and joints, of a human. The marker-free approach directly estimates human posture without the use of markers, which is more convenient for use, though the results may not be as accurate as those of the marker-based approach. This paper considers the marker-free approach. A three-dimensional (3D) posture estimation method that locates 3D significant human body points using only two cameras without any depth sensors is proposed.

Marker-free and vision-based human posture estimation approaches generally include human body segmentation followed by a posture reconstruction process. Automatic segmentation of the human body from a background is indispensable for automatic posture estimation. One commonly used segmentation approach is background subtraction, where the background model is registered and subtracted from each current image for foreground object segmentation. Most human body segmentation algorithms use a gray-level model to distinguish background from moving objects [10, 23–25]. As color cameras are becoming more and more popular and cheaper, several object segmentation algorithms using color models have been proposed [26]. Previous studies have proposed segmentation using the red, green, and blue (RGB) color model [27] and the normalized RGB color model [28]. These color models are sensitive to lightness effects and cannot efficiently reduce the influence of shadow. To address this problem, the brightness component that projects the observed color to the expected chromaticity line is considered in a previous study [29]. However, study [29] does not perform online background registration and update, and therefore, the method cannot be applied to the conditions when there is moving objects in the initial environment. In addition, this method requires the preassignment of four threshold values in segmenting moving objects from background. This paper proposes a linear support vector machine- (SVM-) based method to distinguish background from moving objects. Background is registered based on frame difference and is updated online in this method. Because of its high generalization ability [30], a linear SVM classifier is used to segment moving objects from the background. Not only normalized changes in the RGB channels but also the angle between pixels in the current image and background are fed as inputs to a linear SVM. No thresholds are preassigned in the SVM-based background subtraction step because all parameters in the SVM classifier are automatically determined via learning.

In recent years, object representation using 3D models has become more popular in daily life and has been applied to many areas, such as posture estimation [16–22], object simulation [31], and entertainment [32]. Marker-free 3D human posture estimation is usually performed based on segmented 2D human bodies in different images from multiple cameras. The general approach is to find 2D human silhouette images from different cameras [6, 19–22] and then construct a 3D voxel person based on silhouette volume intersection (SVI). The SVI method is based on the silhouette constraint that a 3D object is encased in the 3D frustum produced by the back projection of a 2D object silhouette image [33–35]. In [6, 21, 22], a 3D voxel person is constructed without identifying significant body parts, which is not suitable for posture analysis or carton animation. In [18, 20], significant body parts are identified based on the 3D voxel person. To obtain a complete 3D body shape, these voxel-person-based estimation methods require at least three cameras and are computationally expensive. For example, 3D body shape is constructed using 25 cameras in [21]. For real-time operation, a personal computer (PC) cluster containing 30 PCs was set up for parallel computing in [21]. Though the Kinect product uses only one camera, an infrared-ray-based depth sensor is required for 3D posture estimation. This paper considers the estimation approach without using any depth sensors.

**Figure 1:**
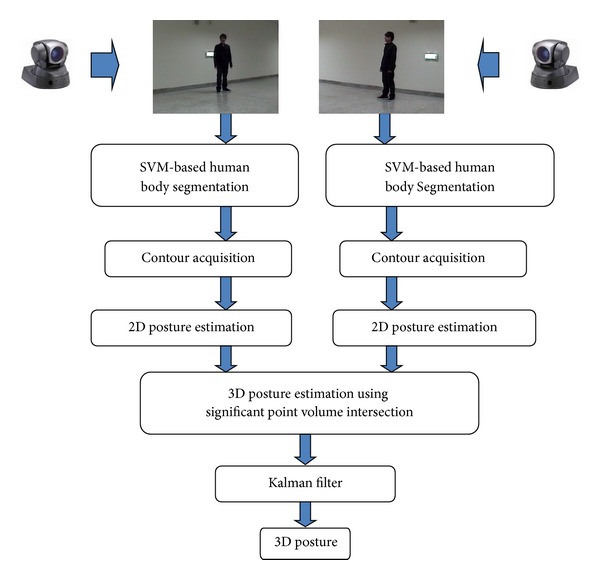
Flowchart of the proposed real-time 3D human model construction method using significant points.

Unlike the voxel-person-based estimation approach above, this paper proposes a new approach that estimates body posture using only two cameras and one PC.  Figure 1 shows a flowchart of the proposed method. After human body segmentation using the SVM-based segmentation method, the proposed approach locates 2D human body significant points, including the body center, the head, the tips of the hands, the tips of the feet, the elbows, and the knees. Two-dimensional locations of the head, the tips of the feet, and the tips of the hands are estimated based on the method proposed in [25] with modifications. The approach in [25] considers 2D localization in static images and uses auxiliary skin color regions to locate the positions of head and tips of hands when shape features alone cannot make a definitive decision. Different from study [25], this paper considers localization in dynamic images and uses estimated locations from previous frames as the auxiliary information when shape features alone cannot make a definitive decision. In addition, this paper proposes a method for locating the elbows and the knees, which was not studied in [25]. Most of all, the located 2D significant points are used to construct significant points in 3D space in this paper, while study [25] considers only 2D localization. This paper proposes a significant point volume matching (SPVM) approach to estimate locations of the significant points in 3D space based on 3D to 2D back projection and matching. The proposed posture estimation method reduces computation time and is suitable for application areas where approximate 3D signification points instead of the whole accurate human body are required, like entertainment and human-robot interaction.

This paper is organized as follows.  Section 2 introduces the SVM-based segmentation method for human body segmentation.  Section 3 describes the 2D human posture estimation approach.  Section 4 introduces the proposed SPVM method for 3D human significant point localization.  Section 5 presents experimental results of the SVM-based segmentation and 2D and 3D human posture estimation. Finally, Section 6 draws conclusions.

## 2. Support Vector Machine-Based Human Body Segmentation Method

This section introduces the SVM-based moving object segmentation method to extract the 2D shape information of the human body from a sequence of images and to reduce the effect of shadows. The proposed method is designed for still cameras. Detailed descriptions of each step are written as follows.

### 2.1. Frame Difference

Let *R*_*n*_, *G*_*n*_, and *B*_*n*_ represent the red, green, and blue color values of a particular pixel in the *n*th frame. For compact notation, the (*x*, *y*) position of the pixel in question is never explicitly noted as all operations are location independent. The difference between the current frame and the previous frame in the RGB color space is calculated to determine whether or not a pixel is stationary. The process can be written as follows:

(1) $$\begin{array}{c} \text{F}{\text{D}}_{n}=\max\left\{\begin{array}{c} \left|R_{n}-R_{n-1}\right|,\\ \left|G_{n}-G_{n-1}\right|,\\ \left|B_{n}-B_{n-1}\right| \end{array}\right\},\\ \text{FD}{\text{M}}_{n}=\{\begin{array}{cc} 1, & \text{if}\;\;\text{F}{\text{D}}_{n}\geq \text{T}{\text{h}}_{\text{FD}}\\ 0, & \text{otherwise}. \end{array} \end{array}$$

If the frame difference FD_*n*_ is smaller than the threshold value, Th_FD_, the corresponding pixel is classified as a stationary pixel and masked using the frame difference mask FDM_*n*_; that is, FDM_*n*_ is set to 0. This paper experimentally sets Th_FD_ to 30. The mask FDM_*n*_ is mainly used to register background. When the threshold is set to a too small value, this step may easily detect a stationary pixel as a moving object and it takes longer time period to construct a complete background. On the contrary, when the threshold is set to a too large value, this step may register part of a moving object as background. Experimental results show that the background registration result is not sensitive to values of Th_FD_ in the range [5, 100].

### 2.2. Background Registration and Update

The goal of this step is to construct reliable background information from video sequences. According to FDM_*n*_, pixels remaining still for a long time in the unregistered background region are considered to be reliable background pixels and registered in the background buffer. This procedure is applied only to the unregistered background region to avoid the registration of a still human body part as a new background. The procedure can be written as follows:

(2) $$\begin{array}{c} \text{S}{\text{I}}_{n}=\{\begin{array}{cc} \text{S}{\text{I}}_{n-1}+1, & \text{FD}{\text{M}}_{n}=0\\ 0, & \text{FD}{\text{M}}_{n}=1, \end{array}\\ \text{IF}\;\;\text{S}{\text{I}}_{n}\geq F_{\text{th}},\;\text{THEN}\{\begin{array}{cc} \mu_{R_{n}}=R_{n} & \\ \mu_{G_{n}}=G_{n} & \\ \mu_{B_{n}}=B_{n}, & \end{array} \end{array}$$

where SI_*n*_ is a stationary index, and *μ*_*R*_*n*__, *μ*_*G*_*n*__, and *μ*_*B*_*n*__ are the *R*, *G*, and *B* components, respectively, of the background buffer value of a pixel in the *n*th frame. The initial values of SI_*n*_ are all set to 0. If a pixel is masked as stationary for *F*_th_ successive frames (i.e., SI_*n*_ > *F*_th_), that pixel is classified as part of the background region. Here, *F*_th_ is set to 30 experimentally based on the speed at which people move indoors. According to our experiments, *F*_th_ may be set at a larger value for detecting an object with a higher moving speed, such as moving people in outdoor environments.

A background pixel in the *n*th frame is modeled by a Gaussian distribution model with means *μ*_*R*_*n*__, *μ*_*G*_*n*__, and *μ*_*B*_*n*__ and standard deviations *σ*_*R*_*n*__, *σ*_*G*_*n*__, and *σ*_*B*_*n*__ in each color component. A registered background buffer pixel is updated using the following equation:

(3) IF {|Rn−μRn|<2σRn, |Gn−μGn|<2σGn,|Bn−μBn|<2σBn}.

Then

(4) μRn=αμRn−1+(1−α)RnσRn2=ασRn−12+(1−α)(Rn−μRn)2μGn=αμGn−1+(1−α)GnσGn2=ασGn−12+(1−α)(Gn−μGn)2,μBn=αμBn−1+(1−α)BnσBn2=ασBn−12+(1−α)(Bn−μBn)2,

where *α*  (0 < *α* < 1) is a predefined constant for temporal averaging. This paper sets *α* to 0.7. The 2*σ*_*R*_*n*__ margin is used and corresponds to roughly 95 percentile inclusion of a normally distributed population.

### 2.3. SVM-Based Object Detection

The SVM-based moving object segmentation method uses a linear SVM classifier to separate the moving object from the background. The normalized background difference (NBD), that is, the difference between the current image and the registered background, in each of the RGB space is extracted as features. The three features used are

(5) $$\begin{array}{c} \text{NB}{\text{D}}_{R_{n}}=\frac{\left|R_{n}-\mu_{R_{n}}\right|}{\sigma_{R_{n}}},\\ \text{NB}{\text{D}}_{G_{n}}=\frac{\left|G_{n}-\mu_{G_{n}}\right|}{\sigma_{G_{n}}},\\ \text{NB}{\text{D}}_{B_{n}}=\frac{\left|B_{n}-\mu_{B_{n}}\right|}{\sigma_{B_{n}}}, \end{array}$$

where standard deviations of each color component in (5) are used to normalize the background difference.

The NBD features consider only the difference in chromaticity and may fail to handle shadow effect. Consider a pixel in the *n*th frame. In the RGB color space, let vectors $\overset{⃑}{V_{o}}=(R_{o},G_{o},B_{o})$ and $\overset{⃑}{V_{b}}=(R_{b},G_{b},B_{b})$ denote RGB color information of this pixel in the segmented object and the background buffer, respectively. The included angle between $\overset{⃑}{V_{\text{o}}}$ and $\overset{⃑}{V_{b}}$ is denoted by *θ*_*i*_. In the RGB color space, if two vectors denote the same color with different lightness, the included angle *θ*_*i*_ between both should be zero, and the distance between both should be nonzero. To measure the difference in lightness, the included angle *θ*_*i*_ is considered in segmentation. The value of cos⁡*θ*_*i*_ is used as another segmentation feature, where

(6) $$\begin{array}{c} \cos\left(\theta_{i}\right)=\frac{{\overset{⃑}{V}}_{o}\cdot \overset{⃑}{V_{b}}}{\left|{\overset{⃑}{V}}_{o}\right|\left|{\overset{⃑}{V}}_{b}\right|}. \end{array}$$

The four-dimensional segmentation feature vector (NBD_*R*_*n*__, NBD_*G*_*n*__, NBD_*B*_*n*__, cos⁡*θ*_*i*_) is fed as inputs to a linear SVM to give a robust segmentation result. The decision function of the linear SVM is

(7) BDMn=sign⁡(w1·NBDRn+w2·NBDGn    +w3·NBDBn+w4·cos⁡⁡(θi)+b).

The output BDM_*n*_ of the SVM is equal to “1” or “−1” when the pixel belongs to a “moving object” or “background,” respectively. The parameters in the decision function (7) are automatically determined through SVM learning [30]. This automatic learning approach avoids manual assignment of thresholds for different environments. The linear SVM is used for computation efficiency. Nonlinear kernel-based SVMs, type-1 and type-2 neural fuzzy systems [36, 37], or SVM-trained fuzzy classifiers [38, 39] may be employed to improve the segmentation performance at the cost of additional computation time.

### 2.4. After Processing

**Figure 2:**
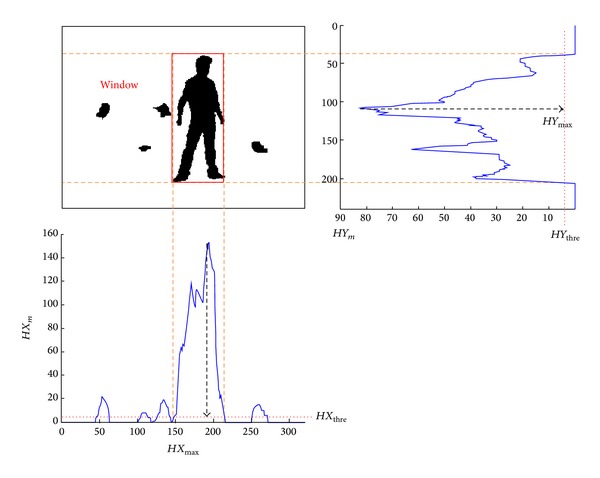
Segmented image, its vertical and horizontal histograms, and position of the found window filter.

After segmentation, the algorithm uses a morphological operator that performs one erosion operation followed by one dilation operation, each of which has 3 × 3 structuring features, to eliminate small noise. Even after the morphological operator, some large noise region remains. To eliminate those regions, this paper uses a rectangular window filter. As shown in Figure 2, the position of the window is determined by horizontal and vertical histograms of the segmented image and histogram thresholds *HX*_thre_ and *HY*_thre_ (both are set to 5 in this paper). Segmented regions outside the window are filtered out. Contours of the regions left are found by using the chain-code algorithm, and the one with the maximum contour length is recorded as the human body. To get a smoother contour, each contour point is replaced by the average of it and its ten forward and backward neighboring contour points. Finally, the silhouette of the body is obtained.

## 3. Two-Dimensional Posture Estimation

Posture estimation is based on the shape of the segmented human body and located significant body points. The details are introduced as follows.

### 3.1. Locating the Head, the Tips of the Feet, and the Tips of the Hands

**Figure 3:**
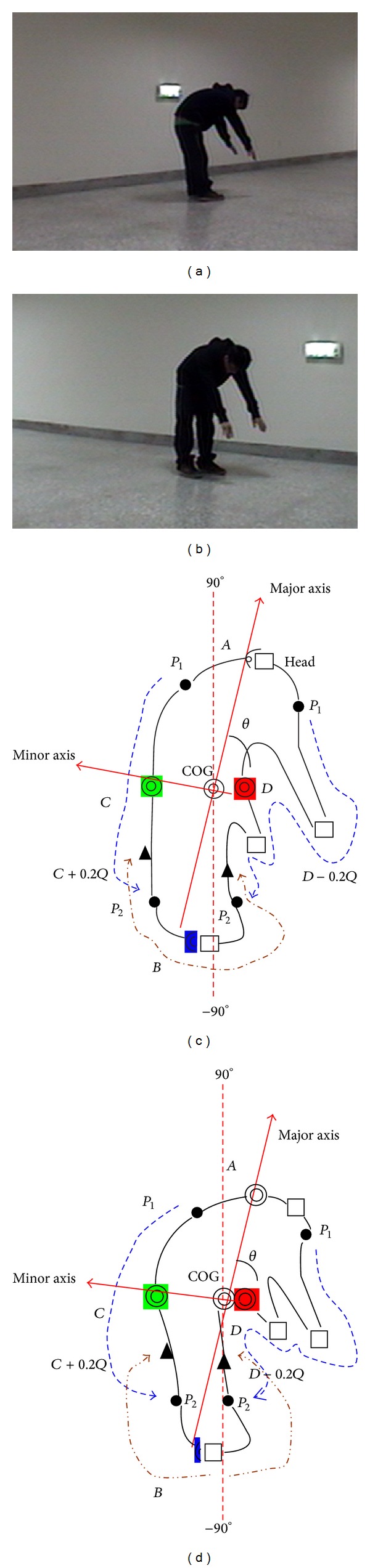
(a) and (b) Original images. (c) and (d) Orientation of the body, directions of the major and minor axes found, their intersection with the body contour, the contour convex points found, and the search regions of the tips of the hands and the feet.

**Figure 4:**
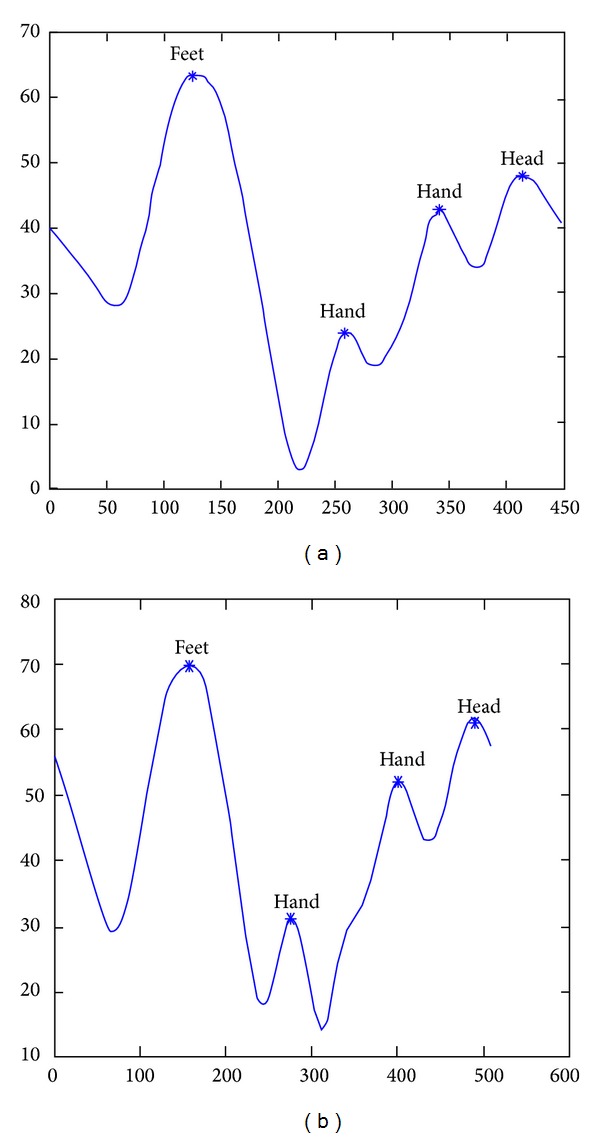
The distance curves of the two images in Figure 3, the local maximum points on the curves, and their corresponding significant body points.

The shape features used for estimation include human body orientation, convex points of the body contours, and convex curvature. The body center of gravity (COG) and orientation are estimated from a best fitting ellipse of the silhouette. Intersections of the major (minor) best-fitting-ellipse axis with the main body contour are denoted by points* A* and* B* (*C* and* D*). Figures 3(a) and 3(b) show two original images taken from two cameras with different perspectives for the same posture. Figures 3(c) and 3(d) show examples of the COG, body orientation (denoted by *θ*), and the major and minor axes and their intersection points with body contours. To find the convex points, the smoothed distance between the COG and each pixel in the contour is computed and a curve is obtained, as shown in Figure 4. The local maxima points in the curve are found to be convex points in the silhouette, as shown in Figures 3(c), 3(d), and 4.

The search region of a significant body point on the contour is determined according to its geometrical location in human body. Signification points usually appear as convex points in a silhouette contour. Therefore, a significant point is selected from the convex point(s) in the search region. When there are multiple candidates, the convex curvature and the geometrical relationship of a convex point with respect to another body point are used to make a final decision. Based on the above idea, determination of a significant point is divided into four steps. The method first locates the head, then the tips of the feet, and finally the tips of the hands. Selection of the values of the thresholds and parameters for search region determination and curvature filtering of each significant point is based on observations of the 2D geometrical structures of human postures. Since the characteristics of each significant point differ, detailed implementation steps for each signification point are different and are described individually.

#### 3.1.1. Locating the Head

**Figure 5:**
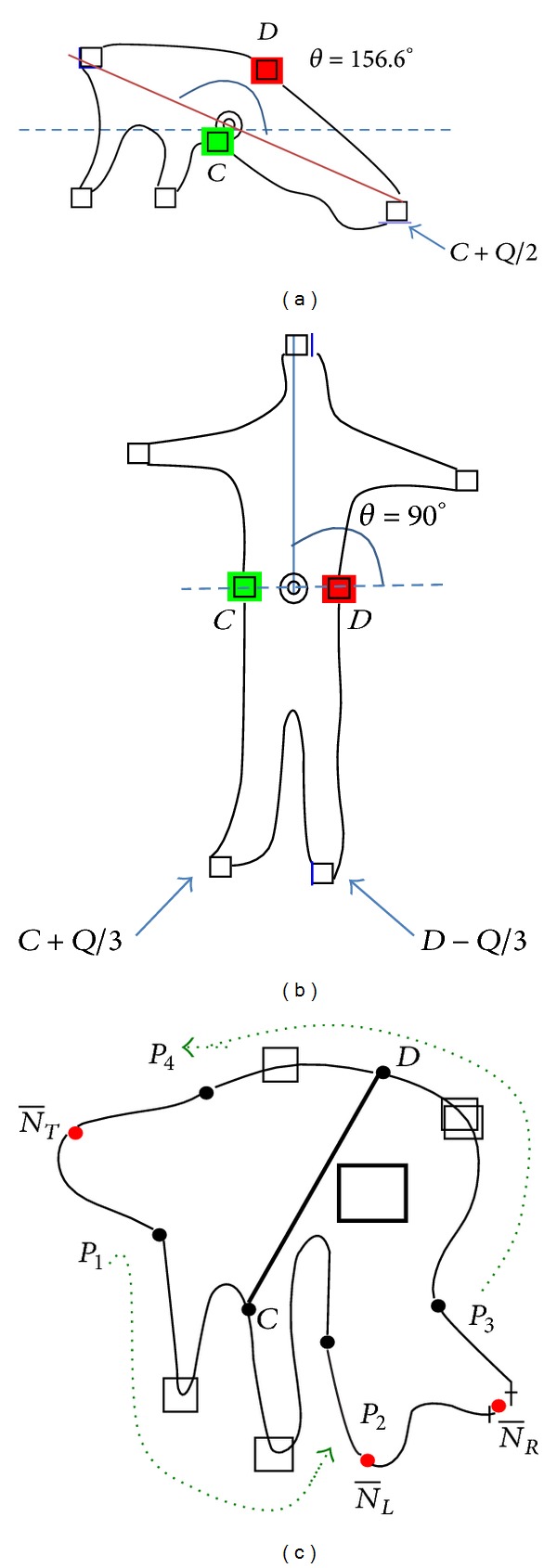
Convex points in different postures: (a) lying, (b) standing, and (c) bending.

*Step 1* (*search region determination*). Locate the head in the identified upper part contour (the part above line CD), when the condition 180 − *θ*_th_ ≥ *θ* ≥ *θ*_th_ is satisfied. This paper sets *θ*_th_ to 15°. Figures 3(a) and 5 show the postures when orientation *θ* = 75.2° and *θ* = 156.6°, respectively. The results in these two figures and other experiments show that when *θ* satisfies the condition, the upper part contour can be correctly identified. When *θ* < 15° or *θ* > 175° (like a lying posture with feet up), the lower part contour may be misidentified as the upper part contour. That is, the foot may be identified as the head. To address this problem, for these conditions, the upper part contour is determined according to the located head position in the previous frame.

*Step 2* (*convex point determination*). Start from point *A* and trace along the contour in both clockwise and counterclockwise directions within the search range. The first convex point found in each direction is a candidate for the head location. If only one convex point is found, the convex point is selected as the head, as shown in Figures 3(c) and 3(d). 

*Step 3* (*curvature filtering*). When two convex points are found, their curvature angles are found. If only one convex point satisfies this angle constraint (within [50°, 150°]), then that point is selected as the head. 

*Step 4* (*geometrical constraint filtering*). If both convex points satisfy or violate the curvature constraint, then the point with the smaller included angle with respect to the major principal axis is selected as the head. Note that if no convex point is found, then point *A* is taken as the head, and the position is further corrected by the Kalman filter algorithm introduced in Section 4.3.

#### 3.1.2. Locating the Tips of the Feet

*Step 1* (*search region determination*). The number of pixels, *Q*, in the lower part of the contour is counted. The search region is the contour points between *C* + *γ* · *Q* and *D* − *γ* · *Q*. This paper sets *γ* to 0.2 with the reason explained as follows.  Figure 5(b) shows the normal posture, where the tips of the feet are approximately located at the points *C* + *Q*/3 and *D* − *Q*/3. That is, the parameter *γ* should be smaller than 1/3 to cover the two tips. In addition, due to body structure constraints, the tips of the feet are seldom near the two interaction points *C* and *D*. Therefore, this paper sets *γ* to 0.2 to discard the noninteresting convex points near the two intersection points.  Figure 3(c) shows that the tip of the left hand, which is near the intersection point *D*, is filtered out by the defined search region.

*Step 2* (*convex point determination*). Find convex points in the search region. 

*Step 3* (*curvature filtering*). Find the convex points whose curvature angle is smaller than 100 degrees. If the number of convex points identified is not greater than two, then these convex points are assigned to be the tips of the feet. Figures 3(c) and 3(d) show that the only convex point found in the search region is identified as the overlapping tips of feet.

*Step 4* (*geometrical constraint filtering*). If the number of convex points identified is greater than two, the two points whose locations are furthest from the head location are selected as the tips of the feet.

#### 3.1.3. Locating the Tips of the Hands

*Step 1* (*search region determination*). Denote the locations of the head and left tip of the foot as $\bar{N_{T}}$ and $\bar{N_{L}}$, respectively. Let $\bar{CD}$ denote the length (number of pixels) between points *C* and *D*. Starting from the head, the left contour search range is the contour pixels between points $P_{1}=\bar{N_{T}}+\lambda \cdot \bar{CD}$ and $P_{2}=\bar{N_{L}}-\lambda \cdot \bar{CD}$. For bending postures, as shown in Figure 5(c), the length of $\bar{CD}$ is generally greater than the other postures such as the standing and lying postures in Figures 5(a) and 5(b). This paper sets *λ* to 0.5 to ensure that the tips of hands are within the search region especially for bending postures, as shown in Figure 5(c). The points in the region with *λ* ∈ [0,0.5) are not considered in order to discard convex points near the head and the tip of the foot. The right contour search range can be found in the same way. Figures 3(c) and 3(d) show that the tips of hands are within the search regions.

*Steps 2 and* *3*. Both of them are identical to those in the feet tips location described above. Figures 3(c) and 3(d) show that the two convex points found in the right search region are identified as the tips of the hands.

*Step 4* (*geometrical constraint filtering*). If the number of remaining convex points is greater than two, then their distances to the body center are compared, and the two points farthest from the center are selected as the tips of the hands. If no convex points are found in the search region, this paper directly designates points *C* and *D* as the tip of the hands.

### 3.2. Locating the Elbows and the Knees

**Figure 6:**
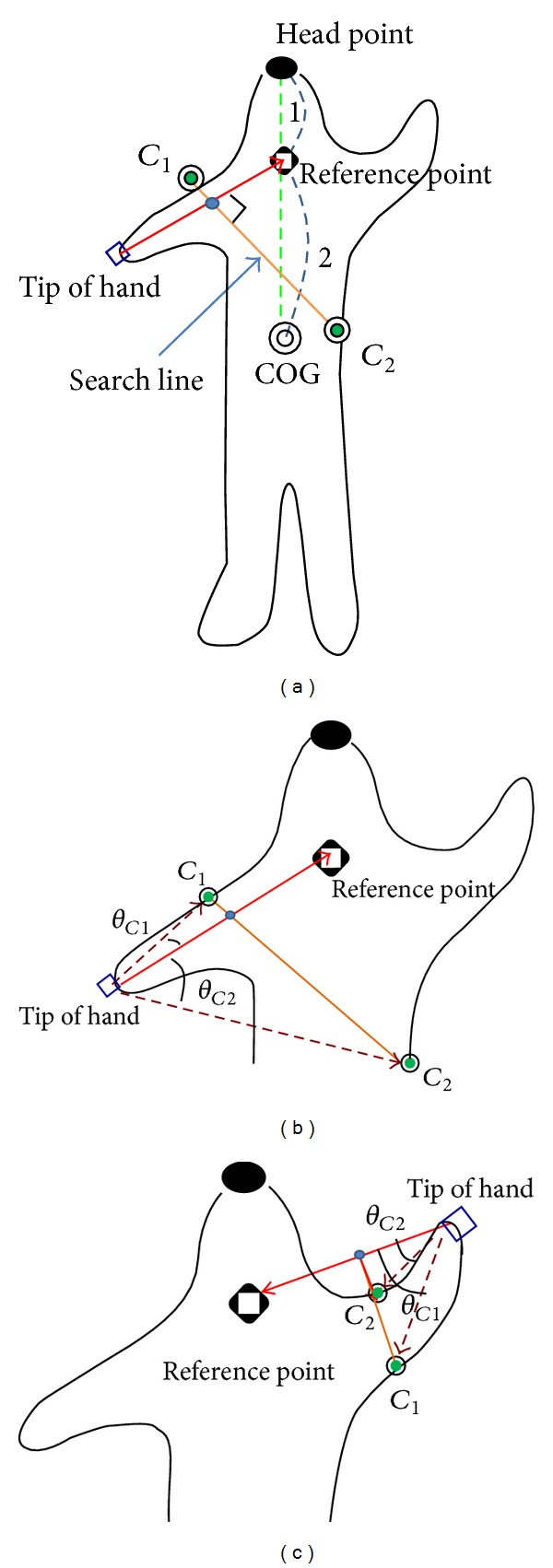
(a) Locations of the two elbow candidates on the contour. (b) and (c) The candidate points *C*_1_ and *C*_2_ and the included angles *θ*_*C*_1__ and *θ*_*C*_2__ for locating the elbows in the hands.

**Figure 7:**
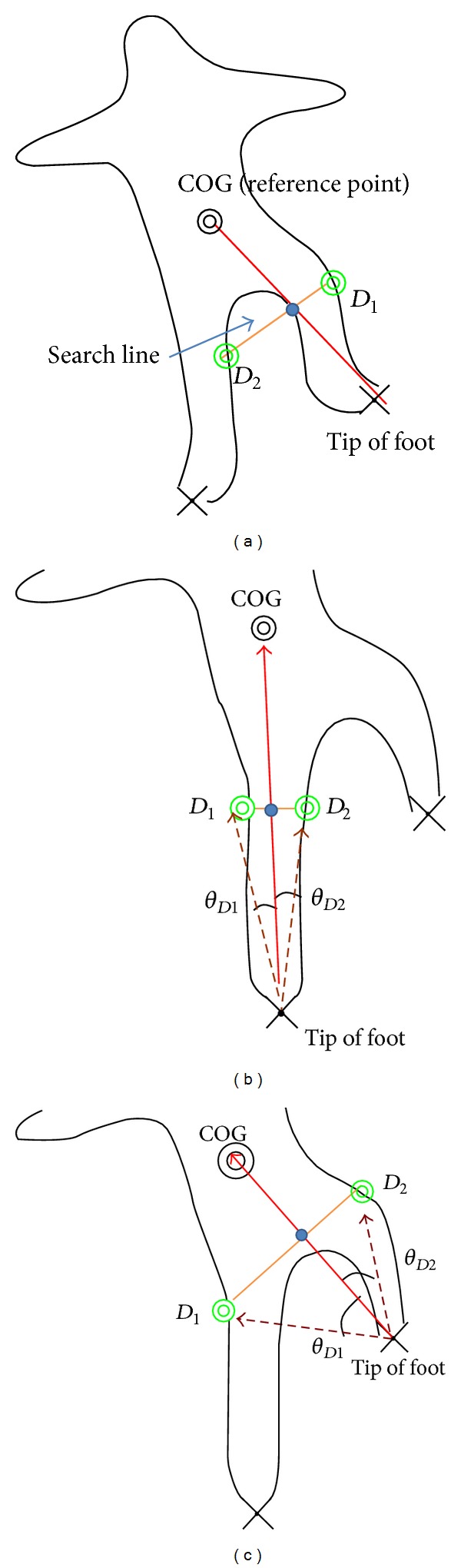
(a) Locations of the two knee candidates on the contour. (b) and (c) The candidate points *D*_1_ and *D*_2_ and the included angles *θ*_*D*_1__ and *θ*_*D*_2__ for locating the knees in the feet.

Estimation of the locations of the elbows (knees) is based on the locations of the tips of the hands (feet). The idea is to find a reference point whose distance to the elbow (knee) is about the same as that from the elbow (knee) to the tip of the hand (foot). This means that the elbow (knee) is located on the search line that is perpendicular to the vector from the tip of the hand (foot) to the reference point and passes through the midpoint of the tip and the reference point, as shown in Figures 6(a) and 7(a). Therefore, the elbow is selected from the intersection points between the search line and the contour. The estimation consists of three steps. The details of each step are introduced as follows.

#### 3.2.1. Locating the Elbows

*Step 1*. Location of the reference point is selected to be near the average of the shoulders. The paper locates the reference point at the line from the head to the COG, as shown in Figure 6(a). Geometrically, the ratio of the distance from the average point of the shoulders to the COG to the distance from the average point to the head is 2 : 1, as shown in Figure 6(a). This ratio is used in locating the reference point at the line from the head to the COG. 

*Step 2*. This step finds candidates of the elbows on the contour. The two intersection points (denoted as *C*_1_ and *C*_2_) of the search line with the contour are candidates of the elbows, as shown in Figure 6(a).

*Step 3*. The included angle between the vectors from the tip of the hand to point *C*_1_ (*C*_2_) and the reference point is denoted as *θ*_*C*_1__ (*θ*_*C*_2__), as shown in Figures 6(b) and 6(c). The point with a smaller included angle is selected as the location of the elbow. In Figure 6(b), point *C*_1_ is selected as the elbow because *θ*_*C*_1__ < *θ*_*C*_2__.

#### 3.2.2. Locating the Knees

The steps for locating the knees are similar to those of locating the elbows and are introduced as follows.

*Step 1*. The COG is used as the reference point. The midpoint between the tip of the foot and the COG is found, as shown in Figure 7(a). 

*Step 2*. This step finds candidates of the knees. The two intersection points (denoted as *D*_1_ and *D*_2_) of the search line with the contour are candidates of the knees, as shown in Figure 7(a). 

*Step 3*. The included angle between the vectors from the tip of the hand to point *D*_1_ (*D*_2_) and the reference point is denoted as *θ*_*D*_1__ (*θ*_*D*_2__), as shown in Figures 7(b) and 7(c). The point with a smaller included angle is selected as the location of the knee. In Figure 7(b), point *D*_1_ is selected as the knee because *θ*_*D*_1__ < *θ*_*D*_2__.

## 4. Three-Dimensional Posture Estimation

A perspective transformation (also called an imaging transformation) projects 3D points onto a 2D plane. The estimation method in Section 3 finds significant points of the human body in the 2D camera coordinate system. The location of a point in the 2D camera coordinate system is denoted by (*x*, *y*)_*C*_. This section introduces how to reconstruct these significant points in the 3D world coordinate system by using the information of two camera images. The location of a point in the world coordinate system is denoted by (*X*, *Y*, *Z*)_*W*_. The proposed method is called significant point volume matching (SPVM). The first step in SPVM is camera calibration in order to find the projection matrices from world coordinate system to different camera coordinate systems. The second is reconstructing the significant points in 3D space based on the idea of silhouette volume intersection [21, 33–35].

### 4.1. Camera Calibration

Assume $\overset{⃑}{V_{I}}\;={\left(M_{X_{I}},M_{Y_{I}},M_{Z_{I}}\right)}_{W}$ is a world-coordinate-system point and $\overset{⃑}{v_{I}}\;={\left(m_{x_{I}},m_{y_{I}}\right)}_{C}$ is the corresponding camera-coordinate-system point. Then the perspective projection from $\overset{⃑}{V_{I}}$ to $\overset{⃑}{v_{I}}$ can be represented by the following equation:

(8) $$\begin{array}{c} W\left[\begin{array}{c} m_{x_{I}}\\ m_{y_{I}}\\ 1 \end{array}\right]=P\left[\begin{array}{c} M_{X_{I}}\\ M_{Y_{I}}\\ M_{Z_{I}}\\ 1 \end{array}\right], \end{array}$$

where *P* is a projection matrix, *W* is a scale factor, and the index *I* denotes the *I*th point. Assume that there are *n* points (*n* ≥ 6) that are noncoplanar in the world coordinate system; *P* is solved using the pseudo-inverse method [40].

### 4.2. Locating 3D Significant Points

The idea of silhouette volume intersection is employed in this paper. The silhouette volume intersection method is based on the silhouette constraint that a 3D object is encased in the 3D frustum produced by the back projection of a 2D object silhouette image. Suppose there are *C* cameras. For the *I*th voxel $\overset{⃑}{V_{I}}\;={\left(M_{X_{I}},M_{Y_{I}},M_{Z_{I}}\right)}_{W}$ in the 3D space, the method uses different projection matrices *P*_*i*_ for channel *i* to obtain its 2D coordinates ${\overset{⃑}{v}}_{I,{\text{ch}}_{i}}$ in images from cameras *i* = 1,…, *C*. The voxel is regarded as part of the object in 3D space if all of the ${\overset{⃑}{v}}_{I,{\text{ch}}_{i}}$ are located in their individual silhouettes.

**Figure 8:**
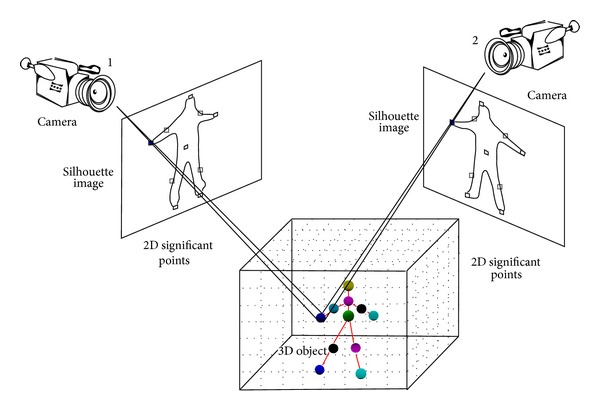
Construction of the 3D location of the tip of a human hand using the SPVM method.

The proposed SPVM method locates different 3D significant points in the world coordinate system using only two sets of 2D camera-coordinate-system significant points from two cameras, that is, *C* = 2. The process of the SPVM for 3D significant point reconstruction is introduced as follows and is illustrated in Figure 8. For each 2D camera-coordinate-system significant point of the human body, a small patch centered at it is defined. Let *R*_*i*,ch_*k*__ denote the patch region, measuring 30 × 30 pixels, that encloses the *i*th significant point of the human body. The indexes *i* = 1,…, 10 denote the ten significant points (body COG, the head, tips of the feet, tips of the hands, the elbows, and the knees), and *k* = 1 and 2 denote cameras 1 and 2, respectively. For a 3D voxel $\overset{⃑}{V_{I}}$, if the two projected points ${\overset{⃑}{v}}_{I,{\text{ch}}_{1}}$ and ${\overset{⃑}{v}}_{I,{\text{ch}}_{2}}$ are located in the 2D patches *R*_*i*,ch_1__ and *R*_*i*,ch_2__, respectively, that define the same *i*th significant point, then $\overset{⃑}{V_{I}}$ is identified as the *i*th significant point.

Suppose that the voxel resolution for the cube in the 3D space is *m* × *m* × *m*. In other words, there are a total of *m* ^3^ voxels. The real size of the cube in the world coordinate system is *S*_*w*_ · (*m* × *m* × *m*), where *S*_*w*_ is a scaling factor. The voxel (*m*/2, *m*/2, 0) in the cube corresponds to the origin (0,0, 0)_*W*_ in the world coordinate system. In other words, the coordinate of the voxel (*I*_*X*_, *I*_*Y*_, *I*_*Z*_) in the cube is *S*_*W*_ · (*I*_*X*_ − *m*/2, *I*_*Y*_ − *m*/2, *I*_*Z*_) when in the world coordinate system. A detailed description of the entire SPVM algorithm is shown in Algorithm 1.

Because there is only a single COG as well as a single head in the body, the index *j* in the patch *R*_*j*,ch_2__ for match checking is set to *i*. For the other significant points, there are left and right candidates in the body. Therefore, for each significant point *i* in camera 1, the two indexes *j*_1_ and *j*_2_ representing the corresponding left and right candidates in camera 2 are selected for match checking. At the end of the SPVM method, multiple 3D voxels $\overset{⃑}{V_{I}}$ may be identified as the same 3D significant point for each (*i*, *j*) pair mainly because of the use of a patch around a significant point. The identification of either (*i*, *j*_1_) or (*i*, *j*_2_) as the actual matching pair for significant point *i* is determined by selecting the one with a larger number of matched voxels. Finally, the average coordinates of the matched 3D voxels are found and used as the final reconstructed position. The SPVM algorithm finds only the 3D localization of each significant point and does not determine whether it is in the left or right hand (foot). The determination that a significant point is in the left or right hand (foot) is made after orientation of the human body is identified. In Figure 8, the 3D position of the tip of human right hand is successfully reconstructed because both of its two projected points in the 2D image planes of cameras 1 and 2 fall in the region of the tip of the right hand.

In addition to the ten reconstructed significant points of the human body, an additional significant point, the chest, is added to construct the human posture. The chest is defined as the midpoint of head and COG, as shown in Figure 8. The SPVM method locates the 3D significant points of a human body instead of a 3D voxel body by the silhouette volume intersection method that does not identify these significant points. In addition, the silhouette volume intersection method may generate many redundant voxels when using a small number of cameras, like the two considered in this paper.

### 4.3. Refinement by the Kalman Filter Algorithm

Finally, this paper applies the Kalman filter algorithm [41] to refine each estimated 3D significant point independently. For the proposed SPVM method, it may happen that no voxel $\overset{⃑}{V_{I}}$ satisfies the projection constraints ${\overset{⃑}{v}}_{I,{\text{ch}}_{1}}\in R_{i,{\text{ch}}_{1}}$ and ${\overset{⃑}{v}}_{I,{\text{ch}}_{2}}\in R_{i,{\text{ch}}_{2}}$ for the *i*th significant point at time step *t* due to inaccurate 2D estimation results. That is, the method fails to find the 3D position in the world coordinate system for the *i*th significant point. For the occasional case, the predicted position using the Kalman filter is used as the 3D significant point position. For the general case that a 3D significant point position $\overset{⃑}{z}(t+1)={\left[\begin{array}{ccc} X^{i} & Y^{i} & Z^{i} \end{array}\right]}^{T}$ is found by the SPVM method, its corrected position from the Kalman filter is used. This Kalman corrector helps smooth the trajectory of a significant point.

## 5. Experiments

**Figure 9:**
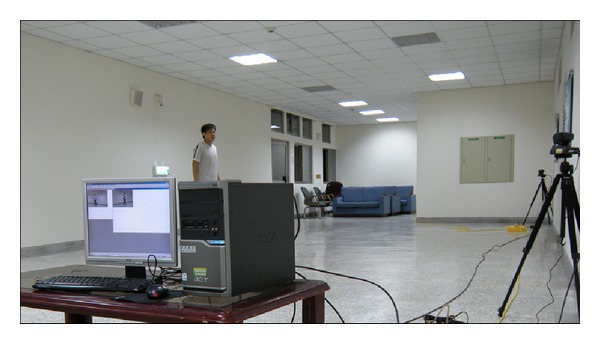
The environment for performing experiments, which shows the locations of the two cameras and the person performing different postures.

In the following experiments, the proposed virtual 3D human model construction system is implemented on a PC with an Intel Core 2 Quad Q6600 2.4 GHz CPU, one “Euresys Picolo Pro 2” image-capture card, and the Windows XP operating system. There are two Sony D100 cameras connected to the image-capture card. Each image measures 320 × 240 pixels, and the program was written in Microsoft Visual C++.  Figure 9 shows the environment for experiments. In the following experiments, the size of each patch *R*_*i*,ch_*k*__ measures 30 × 30 pixels, and the value of *m* for voxel resolution is set to 40. That is, the following experiments use 40 × 40 × 40 voxel resolution under the consideration of computational cost for time-efficient processing. Each voxel corresponds to a 5 cm × 5 cm × 5 cm cube in the real environment; that is, scaling factor *S*_*W*_ equals 5. The processing speed of the entire posture estimation algorithm including human body segmentation is 10 frame/sec for this voxel resolution.

### 5.1. SVM-Based Human Body Segmentation Results

**Experiment 1 (human body segmentation)**

**Figure 10:**
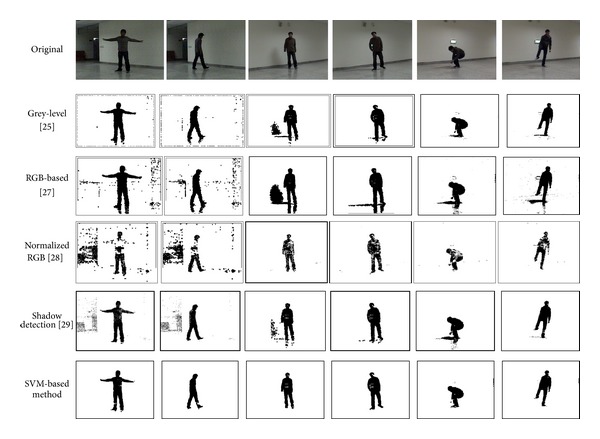
Human body segmentation results of different methods in Experiment 1.

This experiment shows segmentation results of the SVM-based segmentation method. A total of 4000 samples of a moving object (a person), 4000 samples of background, and 2000 samples of the shadow of moving objects were collected as training data set. The training data were collected from an indoor environment. That is, there were 10,000 training samples in total for linear SVM learning.  Figure 10 shows the test segmentation results of the SVM-based segmentation method in the environments different from that used for training. For the purpose of comparison, the morphological operator is applied only to the segmentation results in the last four columns. The results in the first two columns show that there is very little noise in the SVM-based segmentation result even without using the morphological operator.

To verify the performance of the SVM-based segmentation method, segmentation results of different methods are also presented for comparison. These methods include the gray level-based moving object segmentation method [25], RGB-based moving object segmentation method [27], normalized RGB-based moving object segmentation [28], and the shadow detection method in [29].  Figure 10 shows the segmentation results of these methods. For the shadow detection method, different brightness thresholds (*τ*_*α*1_, *τ*_*α*2_) were selected for different environments in order to obtain good results. The thresholds (*τ*_*α*1_, *τ*_*α*2_) were set to (5, −5), (0.1, −0.1), and (0.2, −0.2) for the environments in the first, middle, and last two columns, respectively, in Figure 10. The first two columns in Figure 10 show that there is much noise in the methods used for comparison when the morphological operator is not used. The results show that the normalized RGB segmentation method is very sensitive to small changes in the environment. The gray level- and RGB-based segmentation methods fail to remove the shadow effects. The shadow detection method helps remove shadow effects, but its performance is still worse than the SVM-based method. The SVM-based segmentation method removes most of the shadows and shows better segmentation results than the methods used for comparison.

### 5.2. 3D Significant Point Estimation and Virtual Human Model Construction Results

This subsection presents the posture estimation results of three experiments on standing, bending, sitting, and lying postures.

**Experiment 2 (standing postures)**

**Figure 11:**
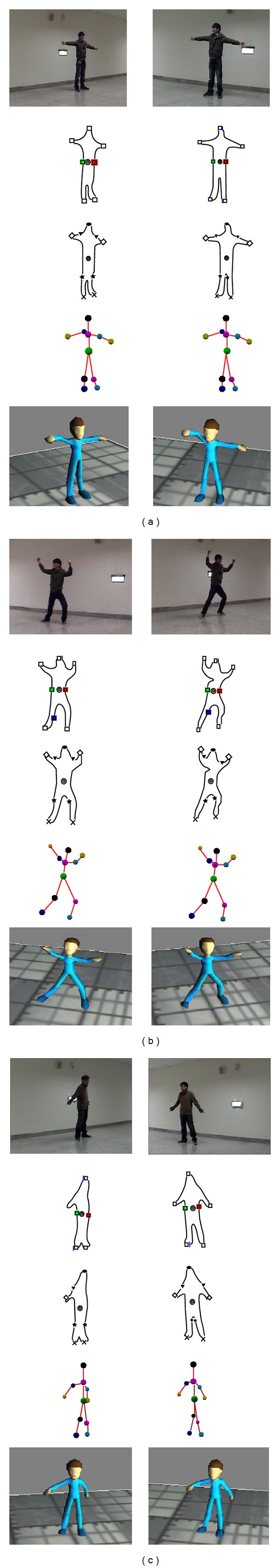
Experimental results of standing postures with hands and/or feet bending in Experiment 2, where notations of the points are listed as follows. 2nd row: convex points on the contour (□), COG (⊚), and the intersection points of the major and minor axes with the contour (■). 3rd row: head (“•”), the tips of the feet (“×”), the tips of the hands (“◊”), the elbows (“▼”), and the knees (“★”).

**Figure 12:**
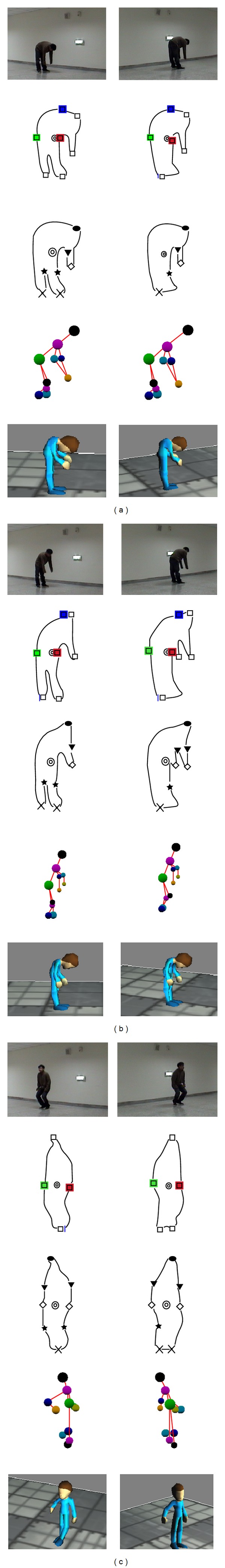
Experimental results of bending postures in Experiment 3, where notations of the points are the same as those in Figure 11.

**Figure 13:**
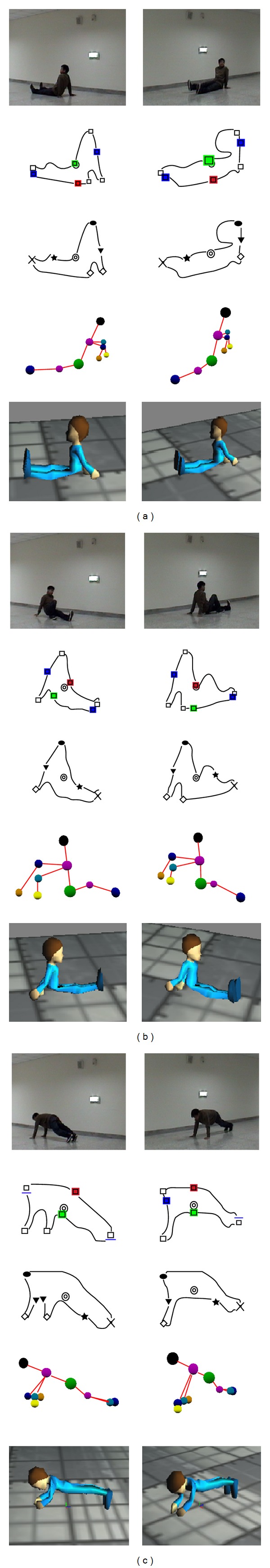
Experimental results of sitting and lying postures in Experiment 4, where notations of the points are the same as those in Figure 11.

Figure 11 shows the experimental results of three different standing postures. In this and Figures 12 and 13, the first row shows a couple of original images from cameras 1 and 2. The second row shows the smoothed contour of a segmented human body, convex points on the contour, COG, and the intersection points of the major and minor axes with the contour. The third row shows the located 2D significant points for each image. The fourth row shows the constructed 3D significant points and skeletons from different perspective views using OpenGL.  Table 1 shows the Euclidean distance errors between the localized significant points and their ground truth obtained via human labeling and measurement. In applications, the estimated 3D significant points can be sent to 3DVIA Virtools software (http://www.3ds.com/products/3dvia/3dvia-virtools/) to construct a virtual 3D character. The last row shows the posture of the constructed virtual 3D character using Virtools.

Figure 11(a) shows that the constructed virtual 3D human model resembles the real human posture with a basic standing posture.  Figure 11(b) shows the experimental result of a standing posture with bending hands and feet. For this posture, localization of the elbows and knees helps construct a more accurate 3D human model. In the left image of Figure 11(c), the tip of the left hand is occluded by another body part, so the system fails to localize it and identifies the tips of the hands as overlapped. In the right image of Figure 11(c), the tip of the left hand is correctly localized. As a result, the SVPI method fails to find the tip of the left hand. The proposed system addresses this problem by using the predicted location from Kalman filtering algorithm. Because the position is obtained via prediction, Table 1 shows that localization error (36.1 cm) of the left hand is relatively larger than those of the other significant points.

**Experiment 3 (bending postures)**

Figure 12 shows the experimental results of three bending postures.  Table 1 shows the Euclidean distance errors between the localized significant points and their ground truth. Overall, Figures 12(a) and 12(b) show that postures of the constructed 3D characters resemble the true postures. Detailed explanations of the points with larger localization errors are given as follows. In the right image of Figure 12(a), the tips of the feet are identified as completely overlapping, and so are the knees accordingly. For this reason, 3D location of one knee is incorrect, as shown in the 3D skeleton. Posture of the 3D character using Virtools considers the true geometric constraint of human body, and therefore, the foot posture does not exactly follow that of the 3D skeleton. In addition, the Virtools draws the direction of the tip of a hand/foot by pointing to a software-defined reference point in the 3D space, which also causes possible dissimilarity between the 3D skeleton and the character.

In each of the two images of Figure 12(a), the system fails to localize the tip of one hand because it is occluded by or occludes another body part. The 2D estimation method identifies the tips of the hands as overlapping in each image. As a result, the estimated 3D locations of the tips of the hands are overlapping, as shown in the 3D skeleton. For this reason, the localization error (30 cm) of the right hand is relatively large.

In the left image of Figure 12(b), the tips of the right hand and the right foot occlude another body part, and so the SVPI method fails to find them. The proposed system localizes it using the predicted locations from the Kalman filter, and therefore, their localization errors (20 cm), as shown in Table 1, are larger than the other points.

In Figure 12(c), the tips of the hands occlude another body part and so no convex points are found in the search regions. The system uses points *C* and *D* as the tips of the hands. The tips of the feet and the knees are identified as overlapping in the left image, and therefore, localization errors (30 cm and 37.4 cm) of the feet are larger than the others in Table 1.

**Experiment 4 (sitting and lying postures)**

Figure 13 shows the experimental results of sitting and lying postures.  Table 1 shows the Euclidean distance errors between the localized significant points and their ground truth. Overall, postures of the constructed 3D characters resemble the real postures. Detailed explanations of the points with relatively larger localization errors are given as follows. For the tip of the right foot in Figures 13(a) and 13(b), the localization error is larger than 40 cm due to occlusion with another body part. Similarly, for the tip of the left hand in Figure 13(b), the localization error is larger than 50 cm due to occlusion with another body part. In the right image of Figure 13(c), the left hand is not correctly segmented, and so the localization error is also larger than 50 cm. As stated above, because of the drawing technique of the Virtools, the gesture of the 3D character does not exactly follow that of the 3D skeleton.

Table 1 shows that both the average localization error and standard deviation (STD) of the head are smaller than those of the other significant points. The localization error of the head in Figure 12(c) is larger than those of the heads in the other figures because part of the head region is not properly segmented in the left image, causing an inaccurate localization of the convex point and the 3D point. It is more difficult to locate the tips of the feet in the sitting posture than in the other postures because occlusion is more likely to occur in the sitting posture, as shown in Figures 13(a) and 13(b). This explains why the tip of the right foot in Figures 13(a) and 13(b) shows a much larger localization error than in the other figures. Overall, the results in Experiments 2–4 show that when occlusion of a significant point with the body occurs, the system may fail to accurately localize that point.  Table 1 shows that the average location errors of the five significant points in the lying and sitting postures are larger than the standing and bending postures. The main reason is that occlusion between the tips of the hands/feet and another body part occurs more likely in the sitting/lying posture than in the other two postures.

**Experiment 5 (comparison with the SVI method)**

**Figure 14:**
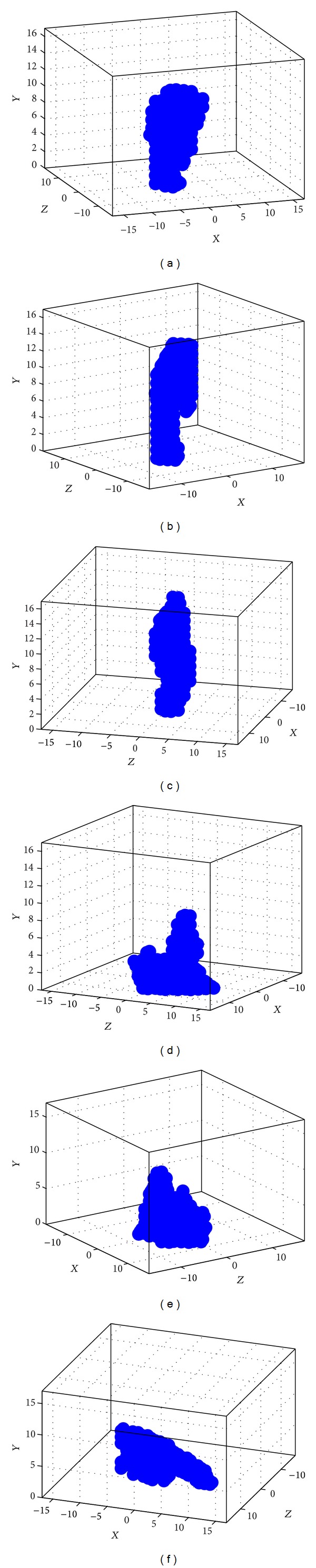
The constructed 3D voxel persons using the original images in Figures 12 and 13 and the SVI method.

For the sake of comparison, Figure 14 shows the constructed 3D voxel persons using the original images in Figures 12 and 13 and the SVI method [6, 18, 21]. The result shows that there are many redundant voxels in the constructed person when using only two cameras. The SVI method needs more cameras to find an accurate voxel person. In addition, this method only constructs a 3D body region and does identify the significant points.

## 6. Conclusions

This paper proposes a 3D human posture estimation method using significant body points. The new techniques of SVM-based segmentation, localization of the knees and the elbows, and SPVM for 3D point reconstruction are proposed in the implemented system. For human body segmentation, the SVM-based segmentation method helps reduce shadow influences so as to obtain a more complete body contour and improve 2D posture estimation results. The experimental results show that the 2D-based 3D significant-point locating method is effective for estimating human 3D posture. By a sequence of concatenated images from two cameras, the 3D estimation method, together with the Kalman filter algorithm, locates an approximate significant point even when there is loss of some significant points by the SPVM method. The following improvements of the system will be studied in the future. In complex environments where the color values of background pixels are very similar to those of moving objects, the proposed segmentation method may fail to obtain a compact object silhouette. The incorporation of motion estimation information into the SVM-based segmentation method will be studied to address this problem. The rules for extracting the significant points are feasible for general postures but may fail for some specific postures with occlusions. To address this problem, incorporation of motion estimation information into the 2D signification point localization approach will also be studied. In addition, localization accuracy of the system can be improved if the kinematic model of the human body is considered at the cost of additional computation load. In future application, the system will be connected to a humanoid robot so that the robot can mimic real human posture in real time.

## Figures and Tables

**Algorithm 1:**
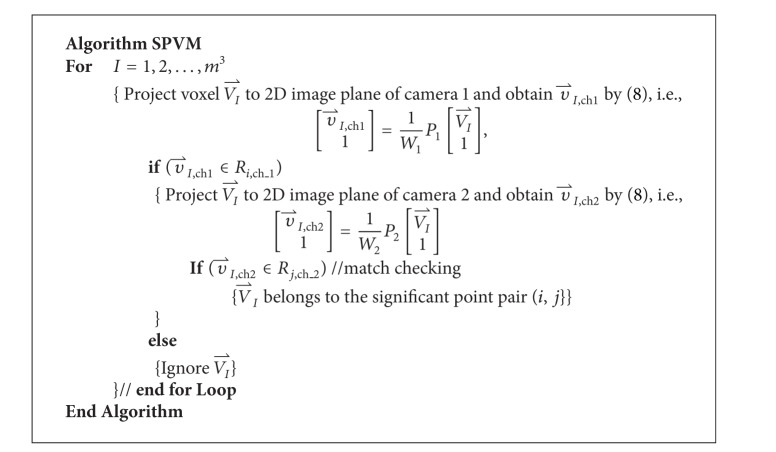


**Table 1:** The localization errors (cm) Of different significant points in Experiments 2–4.

	Head	Tip of right hand	Tip of left hand	Tip of right foot	Tip of left foot	Average error (cm)
Figure 11(a)	2.4	17.3	17.3	3.7	14.1	11.0
Figure 11(b)	14.1	10	10	14.1	36.1	17.9
Figure 11(c)	10	2.1	36.1	3.4	3.4	11.0
Figure 12(a)	10	30	14.1	22.4	10	17.3
Figure 12(b)	10	20	17.3	20	2.7	14.0
Figure 12(c)	22.4	17.3	14.1	30	37.4	24.2
Figure 13(a)	10	22.4	22.4	42.4	14.1	22.3
Figure 13(b)	17.3	31.6	51.0	60.8	1.9	32.5
Figure 13(c)	10	17.3	51.0	20	14.1	22.5

Average error (cm)	11.8	18.7	25.9	24.1	14.9	19.08
Standard deviation (cm)	5.29	8.60	15.11	17.31	12.61	6.58
